# LncRNA MALAT1 Facilitates Ovarian Cancer Progression through Promoting Chemoresistance and Invasiveness in the Tumor Microenvironment

**DOI:** 10.3390/ijms221910201

**Published:** 2021-09-22

**Authors:** Tsui-Lien Mao, Ming-Huei Fan, Nhlanhla Dlamini, Chao-Lien Liu

**Affiliations:** 1 Department of Pathology, College of Medicine, National Taiwan University, Taipei 10002, Taiwan; tlmao@ntu.edu.tw; 2 Department of Pathology, National Taiwan University Hospital, Taipei 10002, Taiwan; 3 School of Medical Laboratory Science and Biotechnology, College of Medical Science and Technology, Taipei Medical University, Taipei 11031, Taiwan; m609104006@tmu.edu.tw; 4 PhD Program in Medical Biotechnology, College of Medical Science and Technology, Taipei Medical University, Taipei 11031, Taiwan; nhlanhlanbs@gmail.com

**Keywords:** metastasis-associated lung adenocarcinoma transcript 1 (MALAT1), epithelial ovarian cancer (EOC), overexpression, chemoresistance, cancer-associated fibroblast (CAF), tumorigenesis

## Abstract

Upregulation of metastasis-associated lung adenocarcinoma transcript 1 (MALAT1, also known as nuclear-enriched abundant transcript 2 (NEAT2) or LINC00047) was found in various solid tumors, including epithelial ovarian cancer (EOC). MALAT1 is a long noncoding (lnc)RNA that regulates many functional signaling pathways, including tumorigenesis. Herein, we observed the consistent upregulation of MALAT1 in MYST4-overexpressing cell lines, while MALAT1 was frequently found to be upregulated in various types of clinical carcinoma tissues, especially EOC. To further investigate the lncRNA MALAT1 in EOC progression, the transduced overexpression of MALAT1 in EOC cell lines and cancer-associated fibroblasts (CAFs) was employed. We found that MALAT1 overexpression in EOC cell lines significantly increased drug resistance, cell migration, and invasion. Furthermore, the concomitant overexpression of MALAT1 in EOC cells and CAFs dramatically increased EOC cell invasion. Accordingly, a mechanistic investigation of MALAT1 overexpression in EOC cells showed that expressions of the cytokines interleukin (IL)-1β and p-P38/p-NFκB/Cox2/prostaglandin E2 (PGE2) signaling were significantly increased, which stimulated inflammatory responses, whereas cell apoptosis was inhibited due to increased Bcl-2 levels and reduced Caspase3 levels. After MALAT1 was overexpressed in EOC cells, and the cyclin D1, p-PI3K, and p-Akt expressions increased, suggesting the promotion of tumor cell proliferation, while increased zinc finger E-box-binding homeobox-2 (ZEB2), yes-associated protein (YAP), and vimentin expression with E-cadherin downregulation indicated the enhancement of the epithelial-to-mesenchymal transition (EMT) in terms of metastasis, thereby triggering EOC progression. Together, our findings demonstrate how MALAT1 overexpression facilitates an oncogenic function through inhibiting tumor cell apoptosis, combined with increasing tumor cell inflammation, proliferation, and invasion in the EOC tumor microenvironment. MALAT1 is thus a potential diagnostic marker and therapeutic for this malignancy.

## 1. Introduction

Regulation of lncRNAs has attracted crucial interest in omics studies in recent years, yet there are still varying opinions as to how these functionally conserved transcripts of more than 200 nucleotides (nt) drive tumorigenesis. These conserved transcripts of noncoding DNA sequences contribute to multiple signaling pathways and functionally interact with many different genes [1]. Furthermore, lncRNAs without a protein-encoding capacity regulate gene expressions through various normal physiological processes, including the regulation of chromatin modification, transcription, and post-transcription [2,3]. MALAT1, a nuclear-restricted housekeeping regulatory lncRNA located on chromosome 11q13, was reported to be overexpressed across various human carcinoma types. Once downregulated, MALAT1 effectively leads to the suppression of tumor cell survival, proliferation, the EMT, and cell migration [4,5,6]. Epithelial ovarian cancer (OC; EOC) is one of the lethal gynecological cancers, with evidence that lncRNAs participate in tumorigenesis [7] and genetic regulatory functions [8,9]. However, diverse functional capabilities of the regulatory mechanisms of MALAT1 have been discovered using different in vitro and in vivo approaches for EOC; these capabilities need to be revealed, and how the aberrant expression of MALAT1 propels EOC progression needs to be emphasized.

The lncRNA MALAT1 was initially found to be highly expressed in metastatic non-small-cell lung cancer [10], while others revealed similar results in different cancer types, including EOC, renal cell carcinoma (RCC), and esophageal squamous cell carcinoma, and it was determined to affect different functional genes [7,11]. Previous studies in lung cancer and RCC have shown that MALAT1-deficient cells lacked the ability to promote tumorigenesis due to decreased cellular proliferation and invasiveness [11,12]. Complex epigenetic regulatory mechanisms involving the histone acetyltransferase enzyme, MYST4 in EOC demonstrated that signaling connecting MYST4 with MALAT1 affects tumor cell proliferation and metastasis in vitro, revealing MALAT1’s oncogenic traits [13,14,15]. In addition to this discovery, evidence regarding the downregulation of MALAT1 expression in vitro compromised the cytoplasmic translocation of heterogeneous nuclear ribonucleoprotein (hnRNP) C and resulted in G_2_/M phase arrest, which showed the participation of MALAT1 in cell cycle regulation [16,17]. Interestingly, the role of MALAT1 in immune regulatory function was suggested by demonstrating that MALAT1 suppressed immunity to chronic infection via promoting Maf/ IL-10 signaling in T helper (Th) cells in mice [18]. Additionally, it was shown that MALAT1 acts as a promoter of inflammation to boost IL-6 production by regulating NF-κB and p38-MAPK signaling pathways in pulmonary microvascular endothelial cells in acute lung injury [19,20]; however, MALAT1 was also reported to be an inflammation inhibitor in the neural microvasculature of ischemic stroke patients by the fact that MALAT1 downregulation significantly induced IL-6 expression and reduced tissue damage [21]. In terms of immunity regulated by the MALAT1 lncRNA, however, pathogenetic manipulation is also impacted by MALAT1 levels. Yong et al. showed that the silencing of MALAT1 resulted in reduced serum levels of IL-6, IL-8, tumor necrosis factor (TNF)-α, skeletal muscle cell apoptosis, and AKT-1 phosphorylation, indicating a promising therapeutic option for sepsis [22]. Furthermore, immune-mediated atherosclerosis was reported to be directly caused by a deficiency of the MALAT1 lncRNA in apolipoprotein E (ApoE)-/- mice [23].

Other evidence regarding the carcinogenic role of MALAT1 in various cancers have been reported [24,25,26] by its functioning as a competitive endogenous (ce)RNA to propel the EMT and metastasis. In addition, networks regulating MALAT1 as a ceRNA in the pathogenesis of EOC showed that MALAT1 enhanced inhibition of apoptosis and promoted DNA synthesis of EOC cells in vitro through the MALAT1/miR-506/inhibitory member of the apoptosis-stimulating protein of p53 (iASPP) axis [27]. Moreover, MALAT1 further facilitates EOC progression by inhibiting miR-211 combined with upregulating PHD finger protein 19 (PHF19) through the MALAT1/miR-211/PHF19 axis [28]. The lncRNA MALAT1 also participates in other mechanisms of EOC tumorigenesis, as it directly interacts with YAP, suppressing its nuclear-cytoplasm translocation and increasing the stemness capacity of EOC cells, while MALAT1 also increases its activity via binding to the Notch1 protein in promoting EOC progression [29,30]. Even though there is accumulating evidence as to the carcinogenic roles of MALAT1, in contrast, Kim et al. has recently shown opposite results, and has indicated that the MALAT1 lncRNA suppresses the progression and metastatic ability of breast cancer cells [31]. Considering the discrepant results regarding the function of MALAT1 in tumorigenesis, more fundamental studies to further investigate the role of the MALAT1 lncRNA in EOC are needed.

In this study, we conducted experiments to explicate the functions of MALAT1 in EOC progression in the tumor microenvironment (TME). First, MALAT1 expression levels in different clinical carcinoma tissues and cancer cell lines were investigated. Next, two EOC lines, HTB75 and OVCAR3, and one CAF (CAF02) cell line were used to overexpress MALAT1. Wound-healing, transwell-invasion, and drug-resistance assays were performed to evaluate tumor cell progression capacities. Finally, the functional elucidation of MALAT1′s regulatory effects in different signaling pathways revealed that the stimulation of inflammation/cell proliferation/metastasis, combined with the inhibition of cell apoptosis in the TME, suggested an enhancement of EOC progression. Our discoveries may be of importance in MALAT1-targeted EOC therapeutic applications.

## 2. Results

### 2.1. Relative Expressions between MYST4 and MALAT1, and Levels of MALAT1 in Different Clinical Cancer Tissues

**Figure 1:**
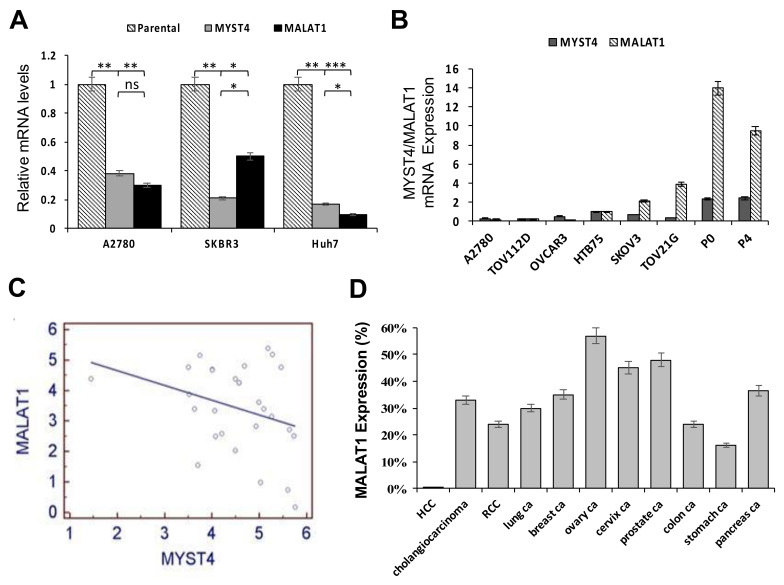
Relative expression levels between MYST4 and MALAT1, and levels of MALAT1 in various types of human cancer tissues: (**A**) Relative expression levels between MYST4 and MALAT1 were detected by an RT-qPCR after MYST4-knockdown in the A2780, Skbr3, and Huh7 cell lines (* *p* < 0.05; ** *p* < 0.01; *** *p* < 0.001). (**B**) Relative expression levels between MYST4 and MALAT1 were measured using an RT-qPCR in the A2780, TOV112D, OVCAR3, HTB75, SKOV3, and TOV21G ovarian cancer (OC) lines and the P0 and P4 endometrial carcinoma cell lines. mRNA expressions were normalized by the level in HTB75 cells. (**C**) Correlated expression levels between *MYST4* and *MALAT1* in 26 ovarian carcinoma tissue samples were calculated using Pearson’s correlation analysis, but no significant correlations (r = 0.3175, *p* = 0.0997) between the two genes were found. (**D**) MALAT1 expression among 11 types of human carcinomas, including hepatocellular (HCC), cholangiolar, renal cell (RCC), lung, breast, ovarian, cervical, prostatic, colon, stomach, and pancreatic carcinomas by in-situ hybridization on tissue microarrays. Ovarian cancer (OC) showed the highest frequency of MALAT1 expression.

Our previous study showed that the MYST4 histone acetyltransferase was significantly overexpressed in ovarian high-grade serous carcinomas (HGSCs) and was correlated with diminished patient survival [13]. Moreover, a significant reduction in MALAT1 mRNA levels was observed in MYST4-knockdown cells by a cDNA microarray analysis, indicating that MALAT1 is a potential downstream target of MYST4. Through an analysis of *MYST4* gene knockdown in cancer cell lines in multiple organs, including the A2780 ovarian cancer cell line, Skbr3 breast cancer cell line, and Huh7 hepatoma cell line, we found significant reductions in MALAT1 levels in the three MYST4-knockdown tumor cell lines compared to their respective parental cells (Figure 1A; * *p* < 0.05, ** *p* < 0.01, and *** *p* < 0.001; Figure S1). Subsequently, relative expression levels of MYST4 and MALAT1 in eight gynecological cancer cell lines, including six OC lines and two endometrial cancer cell lines, were evaluated by a real-time PCR analysis normalized to HTB75 expression. Results showed relatively higher expressions of MALAT1 in the TOV21G, SKOV3, P0, and P4 cell lines, which also exhibited higher expressions of MYST4 (Figure 1B). These data suggested correlated expressions of MYST4 and MALAT1 in gynecological cancers.

Additionally, we further elucidated correlations between MYST4 and MALAT1 expressions in 26 EOC clinical tumor samples by a real-time PCR, as assessed by Pearson correlations. However, in contrast to our previous cDNA array and cell line data, no significant correlations in expression levels between MYST4 and MALAT1 were observed (Figure 1C; r = 0.3175, *p* = 0.0997). This result indicated that complex regulation between both genes may involve some uncertain mechanisms which need to be further investigated. Furthermore, unlike the tumor cell lines, clinical tumor samples included various proportions of non-tumor components in different samples, which may have potentially affected the study results.

Next, to further investigate MALAT1 expression levels in human carcinomas, RNA in-situ hybridization was performed on tissue microarrays containing 11 different human carcinomas from the following organs: liver, bile duct, lung, kidney, breast, ovary, uterine cervix, prostate, colon, stomach, and pancreas. There were 25–32 samples for each tumor, and a positive signal in more than 10% of tumor nuclei was used as a cutoff. EOC exhibited the highest frequency of expression at 55%, whereas hepatomas (HCC) had the lowest frequency of expression at 8% (Figure 1D). Representative MALAT1 expression patterns in human carcinomas from different organs are shown in  Supplemental Figure S2.

### 2.2. MALATI Upregulation in EOCs Stimulates Drug Resistance, Cell Migration, and Cell Invasion In Vitro

To choose cell lines for functional studies of MALAT1 in EOC, comparative expression levels of MALAT1 mRNA were detected by an RT-qPCR among seven EOC cell lines, including SKOV3, HTB75, TOV21G, TOV112D, OVCAR3, OVCAR4, and IGROV1, and four normal human cell lines, including WS1, Hs181.Tes, MRC-5, and Hs67 (Figure S3A), where the MALAT1 level in HTB75 cells was used as the baseline for comparison. Most previous studies investigating the role of MALAT1 in tumorigenesis in vitro were performed by MALAT1-shRNA downregulation assays. In order to further elucidate the role of MALAT1 involved in EOC progression, MALAT1 overexpression using lentiviral transduction in HTB75 and OVCAR3 HGSC cell lines was evaluated in this study. MALAT1 mRNA levels were markedly higher in HTB75 and OVCAR3 cells (*** *p* < 0.001; Figure S3B) transduced with a MALAT1 recombinant lentivirus (MALAT1) compared to the mock-transduced (Mock) and non-transduced parental cells (Parental) controls, as confirmed by an RT-qPCR analysis (Figure S3B), and these were used for further investigations.

**Figure 2:**
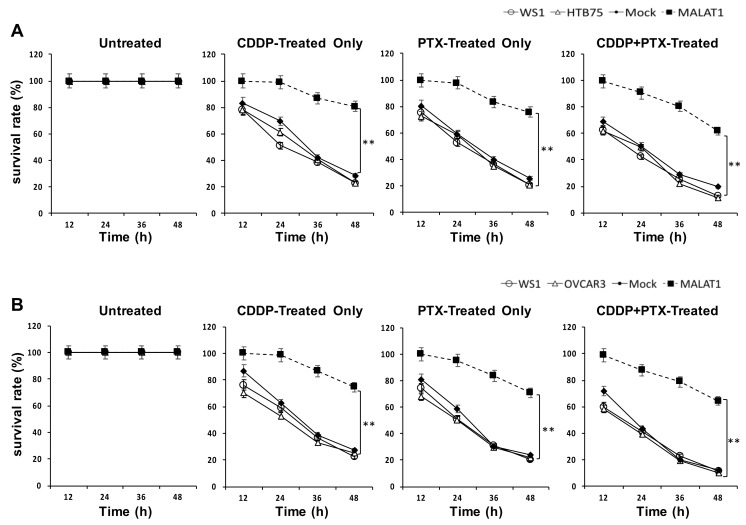
MALAT1 expression contributes to drug-resistance capacities. Effects of MALAT1 overexpression with (**A**) 1.0 μM CDDP alone, 1.2 nM PTX alone, or their combination in HTB75 cells, and (**B**) 1.2 μM CDDP alone, 1.2 nM PTX alone, or their combination in OVCAR3 cells were evaluated by cell viability assays after 12, 24, 36, and 48 h of drug treatment. Data shown were acquired from three independent experiments. ** *p* < 0.01.

**Figure 3:**
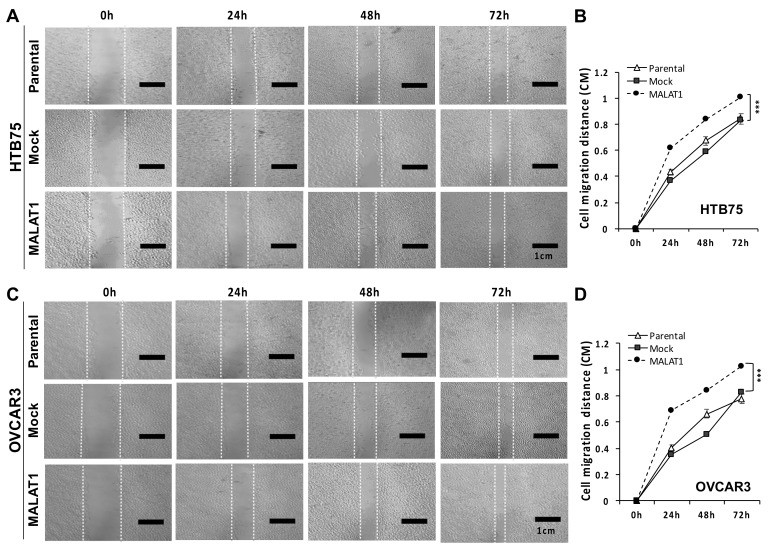
Overexpression of MALAT1 enhanced HTB75 and OVCAR3 cell migration: Representative images of cell migration and their relative migration distances in (**A**,**B**) HTB75 and (**C**,**D**) OVCAR3 cell lines following transduced overexpression of MALAT1. Both MALAT1-overexpressing HTB75 and OVCAR3 cells showed significantly increased migration distances after a wound scratch at 24, 48, and 72 h compared to the mock-transduced (Mock) and non-transduced (Parental) controls (*** *p* < 0.001). The scale bar is 1 cm. Representative experiments were repeated at least three times.

We next investigated the functional role of MALAT1 overexpression in EOC progression. The role of MALAT1 in drug resistance in both HTB75 and OVCAR3 cells was explored after the transduction of MALAT1 (labeled as MALAT1 in Figure 2). In drug-resistance cell viability assays, both HTB75 and OVCAR3 cells overexpressing MALAT1 were more resistant to 1.0 μM cisplatin (CDDP) alone, 1.2 nM paclitaxel (PTX) alone (for HTB75; Figure 2A), 1.2 μM CDDP alone, 1.2 nM PTX alone (for OVCAR3; Figure 2B), and combined CDDP/PTX compared to HTB75/OVCAR3 cells without MALAT1 overexpression (Mock), parental (HTB75/OVCAR3) controls, and the normal human WS1 fibroblast cell line following 12, 24, 36, and 48 h of drug treatment (all ** *p* < 0.01; Figure 2). Collectively, these data confirmed that MALAT1 contributed to drug resistance. Furthermore, in the wound-healing assay, significantly-increased migration extents were found in both HTB75 (*** *p* < 0.001; Figure 3A,B) and OVCAR3 (*** *p* < 0.001; Figure 3C,D) cells overexpressing MALAT1 during incubation periods of 24, 48, and 72 h compared to their respective mock transduction (Mock) and non-transduced parental cell (Parental) controls. These results suggested that MALAT1 overexpression was associated with enhanced tumor aggressiveness in EOCs.

**Figure 4:**
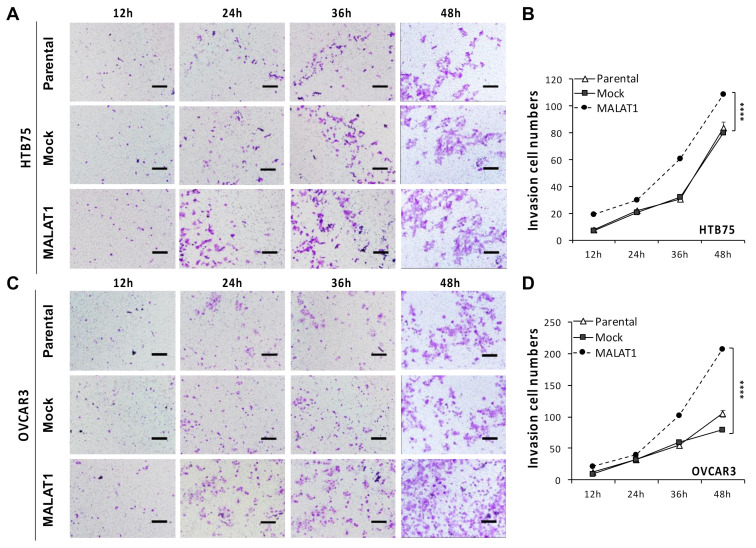
Overexpression of MALAT1 increased the in vitro invasive capacity: Compared to mock-transduced (Mock) and non-transduced (Parental) cells, MALAT1-overexpressing (MALAT1) HTB75 (**A**,**B**) and OVCAR3 (**C**,**D**) cells showed significant increases in invasive cell numbers in the transwell invasive assay (**** *p* < 0.0001). Crystal violet-stained cells were captured after 12, 24, 36, and 48 h of incubation, and the scale bar shown is 1 cm (**A**,**C**). Line figures reveal the invasion cell numbers (**B**,**D**). Data are expressed as the mean ± SD. Similar results were obtained from three independent experiments.

In addition, we also investigated the role of MALAT1 overexpression in EOC progression by transwell assays. Similar results were also obtained from the dramatic increases in cell invasion numbers, as shown in both HTB75 (*** *p* < 0.001; Figure 4A,B) and OVCAR3 cells (*** *p* < 0.001; Figure 4C,D). Invasive cells were counted following 12, 24, 36, and 48 h of incubation where MALAT1-transduced cells (MALAT1) showed significantly increased invasive cell numbers compared to their individual mock-transduced (Mock) and non-transduced parental cells (Parental) controls by crystal violet staining, indicating that MALAT1 overexpression promoted tumor cell invasion in EOCs. Together, these results revealed that elevated MALAT1 levels promoted cell migratory and invasive capacities indicating enhancement of EOC progression.

### 2.3. Overexpression of MALAT1 in CAF02 Fibroblasts Stimulates the In Vitro Invasive Ability of OVCAR3 Cells

**Figure 5:**
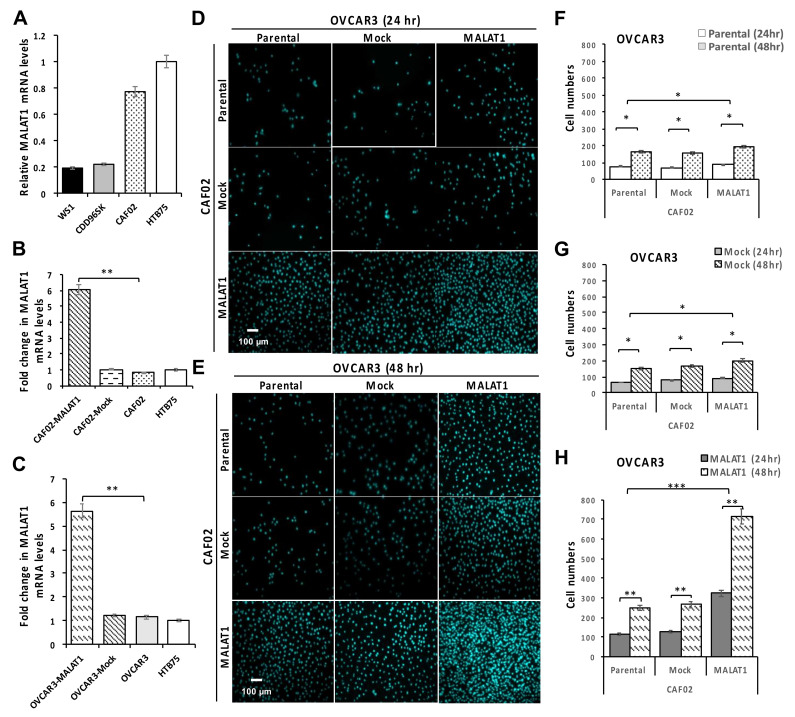
Overexpression of MALAT1 in CAF02 fibroblasts induces OVCAR3 cell invasion: Representative (**A**) original levels of MALAT1 detected by an RT-qPCR analysis. (**B**,**C**) Changes in MALAT1 detected by an RT-qPCR analysis following transduction (**D**–**H**) overexpression of MALAT1 in CAF02 fibroblasts and OVCAR3 ovarian cancer (OC) cells respectively increased OVCAR3 cell invasive abilities (* *p* < 0.05; ** *p* < 0.01; *** *p* < 0.001). The experiment was repeated at least three times.

In the TME, stromal cells such as CAFs are known to be essential to cancer development and progression. To further investigate the role of MALAT1 fibroblasts in OEC cell invasion, the effect of MALAT1 overexpression in CFA02 ovarian CAFs was investigated. Original mRNA levels of MALAT1 in three fibroblast cell lines were measured, with HTB75 cells serving as the expression control (Figure 5A). Compared to individual mock-transduction (CAF02-Mock and OVCAR3-Mock) and parental cells, MALAT1 upregulation in CAF02 (CAF02-MALAT1) and OVCAR3 cells (OVCAR3-MALAT1) showed significantly higher mRNA levels according to an RT-qPCR analysis (Figure 5B,C, * *p* < 0.05 and ** *p* < 0.01, respectively). Following MALAT1 overexpression, the ability of CAF02 cells to promote OVCAR3 invasion was elucidated using an in vitro transwell invasion assay. Compared to parental CAF02 and OVCAR3 cells, the individual upregulation of MALAT1 led to significant increases (Figure 5D–H; * *p* < 0.05 and ** *p* < 0.01, respectively) in the transwell invasive ability of OVCAR3 cells, which indicated that the invasive ability of OVCAR3 cells was closely associated with MALAT1-expressing CAFs and/or EOCs. Interestingly, a dramatically increased OVCAR3 invasive ability was observed with the concurrent overexpression of MALAT1 in both CAF02 fibroblasts and OVCAR3 EOC cells during the 24- and 48-h incubation periods (Figure 5D–H; all ** *p* < 0.01) compared to that of the mock-transduction and parental control groups. These results suggested that MALAT1 is not only important in EOC cells, but is also an important factor in CAFs, particularly in the TME regulating of the invasiveness of EOC cells.

### 2.4. Overexpression of MALAT1 Modulates Tumor Cell Survival by Triggering Functional Molecules Involved in Different Signal Transduction Pathways

**Figure 6:**
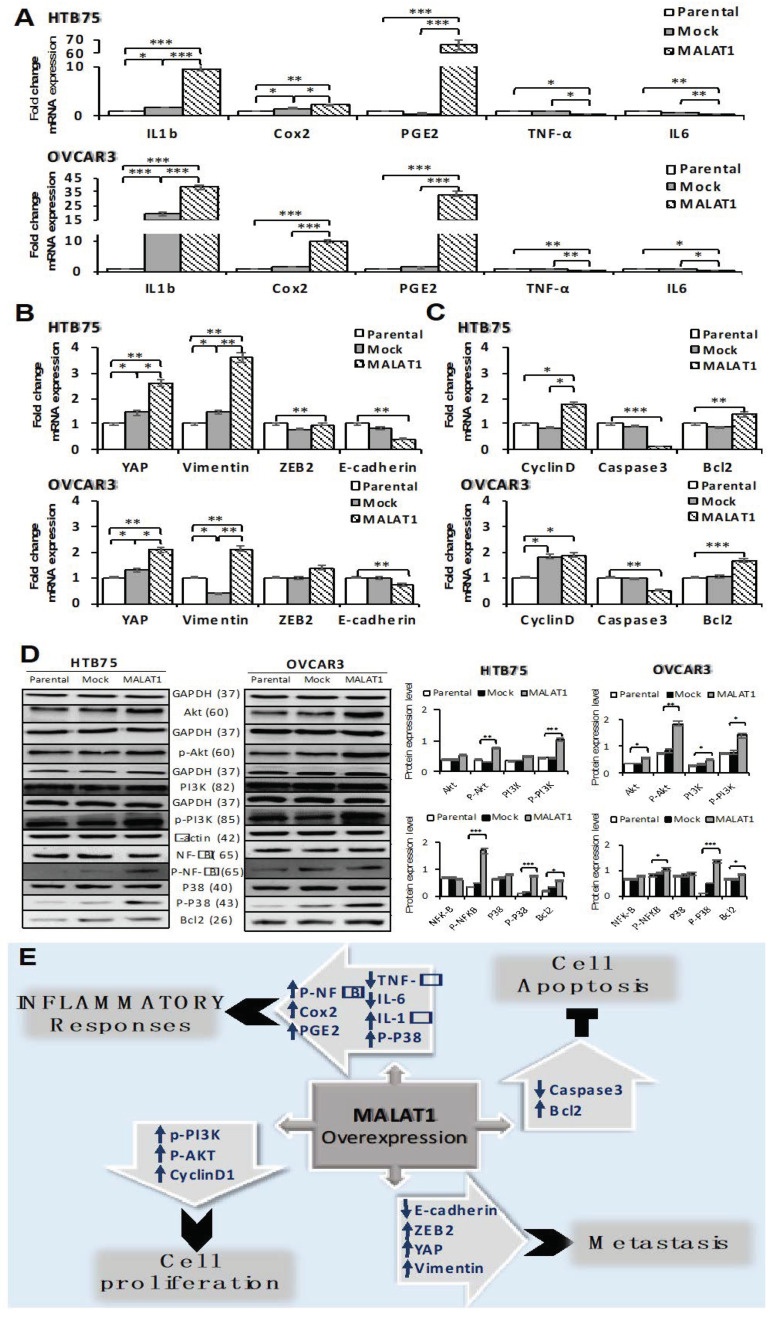
MALAT1 overexpression mediated epithelial ovarian cancer (EOC) progression through enhancing inflammation, cell proliferation, and metastasis, while reducing cell apoptosis: A schematic in vitro model for the overexpression of MALAT1 regulating different functional molecules with similar significance levels in both (**A**–**C**) HTB75 and (**A**–**C**) OVCAR3 cells through upregulation of IL-1β (*** *p* < 0.001), Cox2 (** *p* < 0.01 and *** *p* < 0.001, respectively), PGE2 (***p < 0.001), YAP (** *p* < 0.01), Vimentin (** *p* < 0.01), Cyclin D1 (* *p* < 0.05), Bcl2 (** *p* < 0.01 and *** *p* < 0.001, respectively), and zinc finger E-box-binding 2 (ZEB2) (** *p* < 0.01 and * *p* < 0.05, respectively), and downregulation of tumor necrosis factor (TNF)-α (* *p* < 0.05), interleukin (IL)-6 (** *p* < 0.01), and E-cadherin (** *p* < 0.01) mRNA levels by a real-time PCR analysis. Moreover, (**D**) protein levels of phosphorylated (p)-phosphatidylinositol 3-kinase (PI3K) (*** *p* < 0.001 and * *p* < 0.05, respectively), p-Akt (** *p* < 0.01), p-P38 (*** *p* < 0.001), p-NFκB (*** *p* < 0.001 and * *p* < 0.05, respectively), and Bcl2 (* *p* < 0.05) were also significantly upregulated in MALAT1-overexpressing HTB75 and OVCAR3 cells by a Western blot analysis. (**E**) Functional mechanisms of MALAT1 overexpression in EOC samples include promotion of inflammation, cell proliferation, and metastasis, combined with inhibition of cellular apoptosis, thereby triggering tumor progression in EOCs. Data shown are the mean ± SD of three independent experiments.

To further illustrate the potential mechanisms of the upregulation of MALAT1 in the HTB75 and OVCAR3 EOC cell lines, we evaluated different functional molecules related to cell inflammation, proliferation, metastasis, and apoptosis by an RT-qPCR and Western blot assays. As shown in Figure 6, MALAT1 overexpression in HTB75 and OVCAR3 (Figure 6A–D) cells influenced mRNA expressions of selected molecules with similar significance levels using real-time PCR assays, where MALAT1 overexpression affected inflammatory responses in the TME by upregulating IL-1β, Cox2, and PGE2 (Figure 6A; * *p* < 0.05, ** *p* < 0.01, and *** *p* < 0.001), and downregulating TNF-α (Figure 6A; * *p* < 0.05) and IL-6 (Figure 6A; ** *p* < 0.01). We also investigated cell inflammatory response by examining phosphorylated forms of P38/NFκB signaling using a Western blot assay, which showed significantly increased p-P38 (43 kDa; Figure 6D; *** *p* < 0.001) and p-NFκB (65 kDa; Figure 6D; * *p* < 0.05 and *** *p* < 0.001, respectively) protein levels in MALAT1-overexpressing EOCs compared to the Mock and Parental controls. Moreover, the EMT was also affected by the significant induction of zinc finger E-box binding homeobox 2 (ZEB2), YAP, and vimentin (Figure 6B; ** *p* < 0.01 and * *p* < 0.05, respectively), combined with a reduction in E-cadherin levels (Figure 6B; ** *p* < 0.01). In addition, an obvious effect on cellular apoptosis was observed by Bcl2 upregulation, combined with a reduction in Caspase 3 levels (Figure 6C; ** *p* < 0.01 and *** *p* < 0.001, respectively) in MALAT1-overexpressing EOCs compared to the Mock and Parental controls, in addition to a significant induction of cell cycle regulator cyclin D1 levels in MALAT1-overexpressing EOCs (Figure 6C; * *p* < 0.05) using real-time PCR assay. Furthermore, we also examined the cell-proliferative ability by detecting phosphorylated forms of PI3K/Akt transcription factors using a Western blot analysis, which showed significantly increased p-PI3K (85 kDa; Figure 6D; *** *p* < 0.001 and * *p* < 0.05, respectively) and p-Akt (60 kDa; Figure 6D; ** *p* < 0.01) protein levels in MALAT1-overexpressing EOCs compared to the Mock and Parental controls. Together, overexpression of MALAT1 in EOC cells offered, as aforementioned, favorable conditions for drug resistance, cellular proliferation, migration, and invasion, combined with the inhibition of cell apoptosis in the TME, suggesting the promotion of tumor progression in EOCs.

## 3. Discussion

According to a recent report [32], EOC remains the deadliest cancer of the female reproductive system worldwide, as its 5-year overall survival rate is <30%, due to late diagnosis at an advanced stage with metastasis and also resistance to chemotherapies. The molecular mechanisms of gene regulation, signaling, and metastasis urgently need to be elucidated, including regulatory non-coding (nc)RNAs in the pathogenesis and progression of EOC. lncRNAs, such as MALAT1, were reported to play critical roles in a wide variety of biological processes, including cell differentiation/development, disease pathogenesis, immune responses, and especially cancer progression. Applying in vitro experiments using clinical cancer tissues and cancer cell lines, we observed that overexpression of the lncRNA MALAT1 in EOC cells encouraged drug resistance, invasion, and migration through adaptive remodeling strategies that promote tumor cell survival and proliferation, combined with the inhibition of cellular immunity and apoptosis in the TME, hence triggering EOC tumor progression. Our investigation also revealed that MALAT1 was highly expressed among carcinoma tissues from various organs, including EOCs, as previously reported [26,33,34,35,36,37,38,39]. Mechanistically, the activity of MALAT1 in transcriptional regulation showed that MALAT1-knockdown inhibited the oncogenic function in RCC and consistently depleted histone methyltransferase enhancer of zeste homology 2 (EZH2), a histone lysine N-methyltransferase enzyme (EC 2.1.1.43), resulting in the inhibition of the EMT through the recovery of E-cadherin and a downregulation of β-catenin [40]. Our investigation of network regulatory relationships in cell line studies showed a potential downstream target of the MALAT1 lncRNA when the MYST4 histone methyltransferase was upregulated. Although both showed oncogenic potential when upregulated in EOCs, there was no consistent correlation between MYST4 and MALAT1 expressions in the limited number of clinical EOC samples we examined, suggesting that there may be more complex regulations with uncertain mechanisms which remain to be further uncovered.

Consequently, advanced regulatory roles of MALAT1 in EOC progression were investigated, which included functional signaling molecules that are involved in inflammatory responses, such as the p-P38/p-NFκB/Cox2/PGE2 signaling pathway, and IL-1β inflammatory cytokine, which showed significant upregulation in MALAT1-overexpressing EOC cells. Recent evidence assigned immunoregulatory functions to the MALAT1 lncRNA [19,20,21,22,41], as results showed that a deficiency in MALAT1 resulted in cytokine dysregulation and immune-mediated diseases, such as sepsis and atherosclerosis. Additionally, MALAT1 also acts as an innate immune regulator which modulates innate immune responses by depressing NFκB activity in macrophages and decreases the production of the IL-6 and TNF-α inflammatory cytokines [41]. Moreover, MALAT1-overexpressing dendritic cells (DCs) revealed fewer costimulatory molecules and selectively high inhibitory cytokine IL-10 secretion, resulting in more regulatory T cells (Tregs) with antigen-specific suppression, highlighting that the MALAT1 lncRNA is a novel tolerance regulator of immunity, which has important applications in heart transplantation and autoimmune diseases [42]. In tumorigenic regulation, our findings revealed that upregulation of MALAT1 suppressed immunity via inhibition of the secretion of the IL-6 and TNF-α cytokines, suggesting reduced cellular apoptosis susceptibility within the immunosuppressive TME, thus promoting disease progression in EOC, which is a mechanism thought to result from preventing immune cell infiltration into the TME, promoting tumor cell invasion and migration [43]. As for the more advanced role of MALAT1 involved in immune regulation, further investigation is needed.

Based on an analysis of recent publications on EOC [16], more than 30 lncRNAs act as ceRNAs in EOC progression involved in cell migration, invasion, the EMT, and metastasis. Among these, a number of lncRNAs (such as HOTAIR, NEAT1, and MALAT1) are upregulated as oncogenes and are involved in EOC progression, whereas others are downregulated and act as tumor suppressors (such as GAS5, MIR503HG, and XIST), which are involved in suppressing EOC progression, thus indicating various respective regulatory functions. In the present study, MALAT1 upregulation in EOC cells was correlated with significantly higher levels of p-PI3K, p-AKT, cyclin D1, vimentin, YAP, and ZEB2, combined with a reduction of E-cadherin expression, thus promoting tumor cell proliferation and metastasis. E-Cadherin is responsible for calcium-dependent intracellular interactions and for maintaining the organization of the epithelial cytoskeleton, while ZEB2 is a repressor of E-cadherin and also stimulates the EMT. As previously mentioned, the EMT is associated with metastasis and the acquisition of resistance to chemotherapy. Moreover, stimulation of the EMT is often associated with inhibition of E-cadherin expression and/or with regulation of the PI3K/AKT pathway [16]. Consistent with most previous reports, lncRNA MALAT1-knockdown or -knockout was shown to inactivate the complexes of PI3K/Akt/mammalian targets of rapamycin (mTOR), MAPK/extracellular signal-regulated kinase (ERK), and Wnt/β-catenin signaling pathways [17,44], which significantly suppressed tumorigenicity and induced apoptosis in SKOV3 cells [45] and in other types of cancer cells, emphasizing the specific role of MALAT1 as an oncogenic lncRNA. However, one previous report showed opposite results, and claimed that the MALAT1 lncRNA suppresses metastasis in breast cancer [31], suggesting that further convincing evidence is required to clarify this discrepancy.

In addition to the role of MALAT1 in EOC cells, we also investigated the effect of upregulation of MALAT1 in CAFs. CAFs are an important major cellular component in the TME. We demonstrated that concomitant overexpression of MALAT1 in both EOC cells and CAFs dramatically increased EOC cell invasion compared to the overexpression of MALAT1 in only EOC cells or CAFs. Consistent with our in vitro study, a recent study revealed that MALAT1 was one of nine lncRNAs overexpressed in CAFs microdissected from primary untreated ovarian HGSCs, which were correlated with poorer overall survival [46]. Further functional studies are required to investigate the regulatory mechanisms of MALAT1 in CAFs and the crosstalk with tumor cells in the TME.

Taken together, our experimental results indicated that further studies on lncRNAs in tumor cells and CAFs are required to facilitate the identification of non-coding transcripts such as MALAT1 for targeted biomarker applications. Our study also highlights the need for more experimental clues to prove the crosstalk between the MALAT1 lncRNA and mRNAs/small interfering (si)RNAs or proteins, as well as other cancer hallmark genes involved in cascading tumor cell survival, inflammatory response, proliferation, and the EMT in the TME. Further investigations along these lines could reveal further important information for new insights in MALAT1-induced tumor progression. In summary, our study revealed that MALAT1 overexpression sustained EOC progression in vitro through the regulating of four representative functional mechanisms, suggesting a potential application of MALAT1 as a therapeutic for targeted EOC treatment.

## 4. Materials and Methods

### 4.1. Clinical Samples

Tumor tissue samples from cancer patients with different carcinomas obtained from the liver, bile duct, lung, kidney, breast, ovary, uterine cervix, prostate, colon, stomach, and pancreas were analyzed for MALAT1 and MYST4 expression levels. The study was performed according to approval by the National Taiwan University Hospital-Research Ethics Committee (NTU-REC protocol no.: 201503102RINA, 7 July 2015), and informed consent was obtained from each patient. The research, including the use of biohazards, biological agents, toxins, materials, and reagents, followed standard biosafety regulations.

### 4.2. In Situ Hybridization

Paraffin-embedded tissue sections were cut into samples with a thickness of 4-μm, followed by the detection of MALAT1 expression using the RNAscope^R^ system (Advanced Cell Diagnostics, Hayward, CA, USA) as per the manufacturer’s protocol. RNA quality control was checked by a control probe included in the experimental protocol. Positive staining of MALAT1 was accepted when more than 10% of tumor cells were stained.

### 4.3. Cell Lines and Cultivation

Human EOC cell lines (A2780, HTB75, IGROV1, OVCAR3, OVCAR4, SKOV3, TOV21G, and TOV112D), a breast cancer cell line (Skbr3), a hepatoma cell line (Huh7), and normal human cell lines (WS1, Hs181.Tes, MRC-5, and Hs67) were purchased from American Type Culture Collection (ATCC, Manassas, VA, USA), while human ovarian CAFs (CAF02) were purchased from Neuromics (Edina, MN, USA). Endometrial cancer cell lines (P0 and P4) were kind gifts from Dr. PL Torng (National Taiwan University Hospital, Hsin-Chu Branch). Cell lines were maintained in either Dulbecco’s modified Eagle’s medium (DMEM; Life Technologies, Carlsbad, CA, USA) or Roswell Park Memorial Institute (RPMI) 1640 medium (Gibco, Rockville, MD, USA), supplemented with 10% fetal bovine serum (FBS; HyClone, Logan, UT, USA), 1% antibacterial/antimycotic (AA; Mediatech, Manassas, VA, USA), and 1% HEPES buffer solution (Corning, Corning, NY, USA), and then incubated at 37 °C with 5% CO_2_; the medium was replaced every 2–3 days. All cell lines were tested to identify the species and detect mycoplasma, and cell lines purchased from ATCC were authenticated using a short-tandem repeat (STR) profile analysis by ATCC.

### 4.4. Lentiviral Production

Short hairpin (sh)RNA pLKO.1 vectors were purchased from the RNAi Core Facility of Academia Sinica (Taipei, Taiwan). The pCDH-hMALAT1 lentiviral vector was purchased from Addgene (plasmid no. 118580; Watertown, MA, USA). Briefly, human 293T cells were seeded at 9 × 10^6^ cells/15-cm dish 24 h prior to transfection. All plasmid DNA was purified using an Endo-Free Maxi prep kit (Qiagen, Valencia, CA, USA). 293T cells were transfected with 7.5 μg of an empty vector (mock) or the recombinant plasmid vector in addition to 4.5 μg of the psPAX2 packaging plasmid and 3 μg of the pMD2G vesicular stomatitis virus envelope plasmid using a calcium phosphate transfection system [47]. Viral supernatants were harvested at 48 h after transfection.

### 4.5. MYST4 shRNA Knockdown

To knock down endogenous human MYST4, shRNA pLKO.1 vectors were transfected using a lentiviral system [47]. The shRNA with the highest knockdown effect among the five shRNAs against the MYST4 we tested was selected for further study. The highest effective shRNA sequence of MYST4 was as follows: sh48 (TRCN0000-245348) GATATTAGAAGTCGGTTTATT. An shRNA vector against LacZ was used as a scrambled control. Transduced cells, including A2780, Skbr3, and Huh7 cells, were then selected with puromycin.

### 4.6. In Vitro Overexpression of MALAT1

Human MALAT1 was overexpressed in ovarian cancer cell lines (HTB75 and OVCAR3) and ovarian CAFs (CAF02). All three cell types were individually seeded at a density of 5 × 10^5^ cells/well on 24-well plates using RPMI 1640 medium (Corning). After overnight incubation, 2 mL of fresh media containing 8 μg/mL polybrene (Sigma–Aldrich, St. Louis, MO, USA) and the recombinant lentivirus was added. Following transduction and puromycin (Sigma–Aldrich) selection, all three HTB75, OVCAR3, and CAF02 cells with stable MALAT1 overexpression were used for subsequent assays.

### 4.7. Real-Time Reverse-Transcription Quantitative Polymerase Chain Reaction (RT-qPCR)

Cells were collected and lysed for total RNA extraction using a LabPrep^TM^ RNA Mini Kit (Taigen Bioscience, Taipei, Taiwan). Complementary (c)DNA was synthesized by RT of RNA using a High-Capacity cDNA Reverse Transcription Kit (ThermoFisher Scientific, Foster City, CA, USA) according to the manufacturer’s standard protocols. Briefly, the qPCR assay was performed in triplicate for each cDNA sample on an Applied Biosystems StepOne^TM^ and StepOnePlus^TM^ Real-Time PCR analyzer (Applied Biosystems, ThermoFisher Scientific) using PowerUp^TM^ SYBR^TM^ Green Master Mix (ThermoFisher Scientific). Normalization was performed with the S26 housekeeping gene according to the crossing threshold (Ct) value of the transcripts assessed by the RT-qPCR and calculated using the 2^−ΔΔCt^ method. Changes in messenger (m)RNA expression levels are expressed as multiples of change relative to the control mean ± standard deviation (SD). Primer sequences used are listed in Table 1.

### 4.8. Drug-Resistance Cell Viability Assay

Cell viability was determined with a 3-(4,5-dimethylthiazol-2-yl)-2,5-diphenyltetrazolium bromide (MTT; ThermoFisher Scientific) assay, as previously described [44]. Briefly, cells were seeded into 96-well plates, cultured with complete medium for 16 h, and then treated with certain concentrations of CDDP (1.0 or 1.2 μM; Sigma–Aldrich), PTX (1.2 nM; Sigma–Aldrich), or their combination for various time points. Results of the 50% inhibitory concentration (IC_50_) titration of CDDP/PTX for HTB75, OVCAR3, and their MALAT1-overexpressing cells, are shown in Figure S4. Following removal of the supernatant, DMEM containing 5 mg/mL MTT was added and incubated for 4 h. The resulting formazan was dissolved in 1 mL isopropanol, and the absorbance at 570 nm was measured with a microplate reader (Bio-Rad Laboratories, Hercules, CA, USA). The number of viable cells was proportional to the absorbance, and cell viability was presented as a percentage of the dimethyl sulfoxide (DMSO) control (untreated).

### 4.9. Wound-Healing Assay

The wound-scratch method was used for the cell-migration assay. Cells overexpressing MALAT1, mock-transduced cells, and parental cells were seeded at 2 × 10^5^ cells/well in six-well plates with RPMI complete medium for the experiments until at least 80% confluency. A 200-μL micropipette tip was used to make a scratch wound, and, after rinsing with culture medium, cells were further incubated at 37 °C. Monolayer wound closure was viewed under an inverted microscope, and the migration distance was measured between the wounded edges every 24 h for 3 days.

### 4.10. Transwell-Invasion Assay

The invasion assay was performed using 24-well transwell chambers containing membranes coated with Matrigel (Merck Millipore, St. Louis, MO, USA). Transfected and parental cells were seeded at 2 × 10^5^ cells/well in the upper chamber, which contained serum-free RPMI 1640 medium, whereas RPMI 1640, containing 10% fetal bovine serum (FBS) or CAF02 cells with/without MALAT1 overexpression, were added to the lower chamber. Following 48 h of incubation, cells that had not migrated across the membrane were removed with a cotton swab, while cells that had migrated to the lower surface of the inserts were stained using 2% crystal violet and/or diamidino-2-phenylindole (DAPI) for 10 min. Complete filters were washed twice with water before the observation.

### 4.11. Western Blotting

Cultured HTB75 and OVCAR3 cell lines including parental, mock-transduced, and MALAT1-transduced cells were seeded and collected for Western blotting. Cell lysates were denatured and electrophoresed by 12% SDS-PAGE, as previously described. Primary antibodies against PI3K, phosphorylated (p)-PI3K, Akt, p-Akt, NFκB, p-NFκB, P38, p-P38, Bcl-2, β-actin, and glyceraldehyde 3-phosphate dehydrogenase (GAPDH) were purchased from ElabScience Biotechnology (Houston, TX, USA), and GAPDH/β-actin were used as a loading control. Appropriate secondary antibodies were used to detect proteins, as previously described. An ImageQuant^TM^ LAS 4000 analyzer (GE Healthcare Life Science, Pittsburgh, PA, USA) was used to quantify protein expression levels.

### 4.12. Statistical Analysis

Experimental data are expressed as the mean ± standard deviation (SD) of at least three independent experiments unless otherwise specified. A two-tailed Student’s *t*-test was used for intergroup comparisons. Comparisons between groups were determined with a one-way analysis of variance (ANOVA). Differences were considered statistically significant at *p* values of * *p* ≤ 0.05, ** *p* ≤ 0.01, *** *p* ≤ 0.001, and **** *p* ≤ 0.0001 using GraphPad Prism 8.3.0 software (San Diego, CA, USA). Pearson’s correlation analysis was utilized for the correlation between the expressions of MALAT1 and MYST4.

## Figures and Tables

**Table 1:** Primer sequences used for the RT-qPCR analysis.

Gene Name	Primer Sequences	GenBank No.
MYST4 F	5′ AACCT GTTCC AGAGC CAATG 3′	XM_867653
MYST4 R	5′ TGTAC TTTTG CAGGT GATTG 3′	
MALAT1 F	5′ GGTTG AGATG AAGCT TCTT 3′	NR002819.4
MALAT1 R	5′ GCACT TCTTG TGTTC TCTT 3′	
TNF-α F	5′ TACTG ATACA TACAA CCACG 3′	NT_167244.2
TNF-α R	5′ ATTTT CAGGT CCCCA GCAGG 3′	
IL-6 F	5′ TTGAG GGAAA AGAGT AGAGG 3′	NC_000007.14
IL-6 R	5′ TAGGC TTTCT ATGGT GGTGG 3′	
YAP F	5′ ATCAGATCGTGCACGTCC 3′	AB567721.1
YAP R	5′ ATCAGTACTGGCCTGTCG 3′	
Cox2 F	5′ AGTGCGATTGTACCCGG 3′	AY462100.1
Cox2 R	5′ TTGCATTTCGAAGGAAGG 3′	
PGE2 F	5′ AACCCTGTGCGCAGGGC 3′	NM_198938.3
PGE2 R	5′ TTGCCATTTGGCCTCCC 3′	
IL-1β F	5′ AAGCTGAGGAAGATGCTGG 3′	NM_000576.3
IL-1β R	5′ TTGCTGTGAGTCCCGGAGC 3′	
Cyclin-D1 F	5′ AAGGCGGAGGAGACCTGC 3′	NM_053056.3
Cyclin-D1 R	5′ ATGGCCAGCGGGAAGACC 3′	
Caspase-3 F	5′ TACCT GTGGC TGTGT ATCC 3′	NC_000004.12
Caspase-3 R	5′ AAACT CACTG AAGTG CTGC 3′	
Bcl2 F	5′ TTGTC ATCAG CTCGC TCTCC 3′	XR_935248.3
Bcl2 R	5′ TATCC GGCAC ATGGA GGCGG 3′	
ZEB2 F	5’ AAGTG AGAAG GGGTG TCC 3’	NR149354.1
ZEB2 R	5’ TTTCC TGGGC TGGAT AGC 3’	
E-Cadherin F	5′ TTTCC CTCAA GGCTG CAGG 3′	NC_000074.7
E-Cadherin R	5′ ATGCC AGGAG GCCGC TCTC 3′	
Vimentin F	5′ TAAACCGCTAGGAGCCC 3′	NM_003380.5
Vimentin R	5′ ATGGCTGCGGAGGGTGG 3′	
S26 F	5’ CCGTG CCTCC AAGAT GACAA AG 3’	NM001029.5
S26 R	5’ GTTCG GTCCT TGCGG GCTTC AC 3’	

F, forward; R, reverse.

## Data Availability

The datasets used and/or analyzed during the current study are available from the corresponding author upon a reasonable request.

## Supplementary Materials

The following materials are available online at https://www.mdpi.com/article/10.3390/ijms221910201/s1. Figure S1: Three different cancer cell lines (A2780, SKBR3, and Huh7) were selected for a further MYST4-knockdown investigation; Figure S2: Representative figure of MALAT1 RNA in situ hybridization; Figure S3: MALAT1 expression in various cell lines; Figure S4: Growth inhibition by cisplatin (CDDP) and paclitaxel (PTX) in HTB75/OVCAR3 parental cells (HTB75/OVCAR3) and HTB75/OVCAR3 cells transduced with MALAT1 (MALAT1).
