# Supplementary material for: LncRNA MALAT1 Facilitates Ovarian Cancer Progression through Promoting Chemoresistance and Invasiveness in the Tumor Microenvironment
Source: Int J Mol Sci. 2021 Sep 22;22(19):10201. doi: 10.3390/ijms221910201 (PMC8508663; doi:10.3390/ijms221910201)
Supplement: Supplementary file 1 [file ijms-22-10201-s001.zip › ijms-1348438-supplementary.pdf]

Supplemental Results

09182021

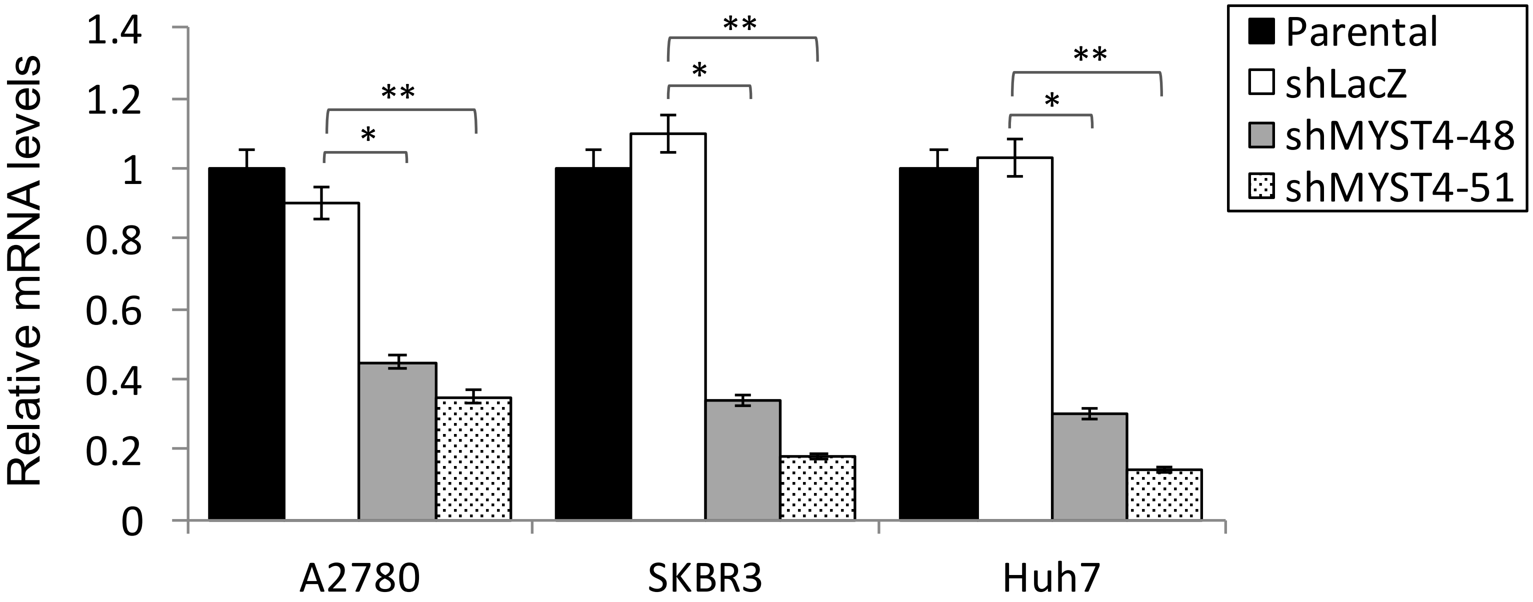

Figure S1. Three different cancer cell lines (A2780, SKBR3, and Huh7) were selected for a further MYST4-knockdown investigation. shMYST4-48 and -51 clones showed significant MYST4 silencing efficacy compared to the control (sh*LacZ*) and parental cells. Data are presented as the mean ± SD of three independent experiments. *P<0.05, **P<0.01.

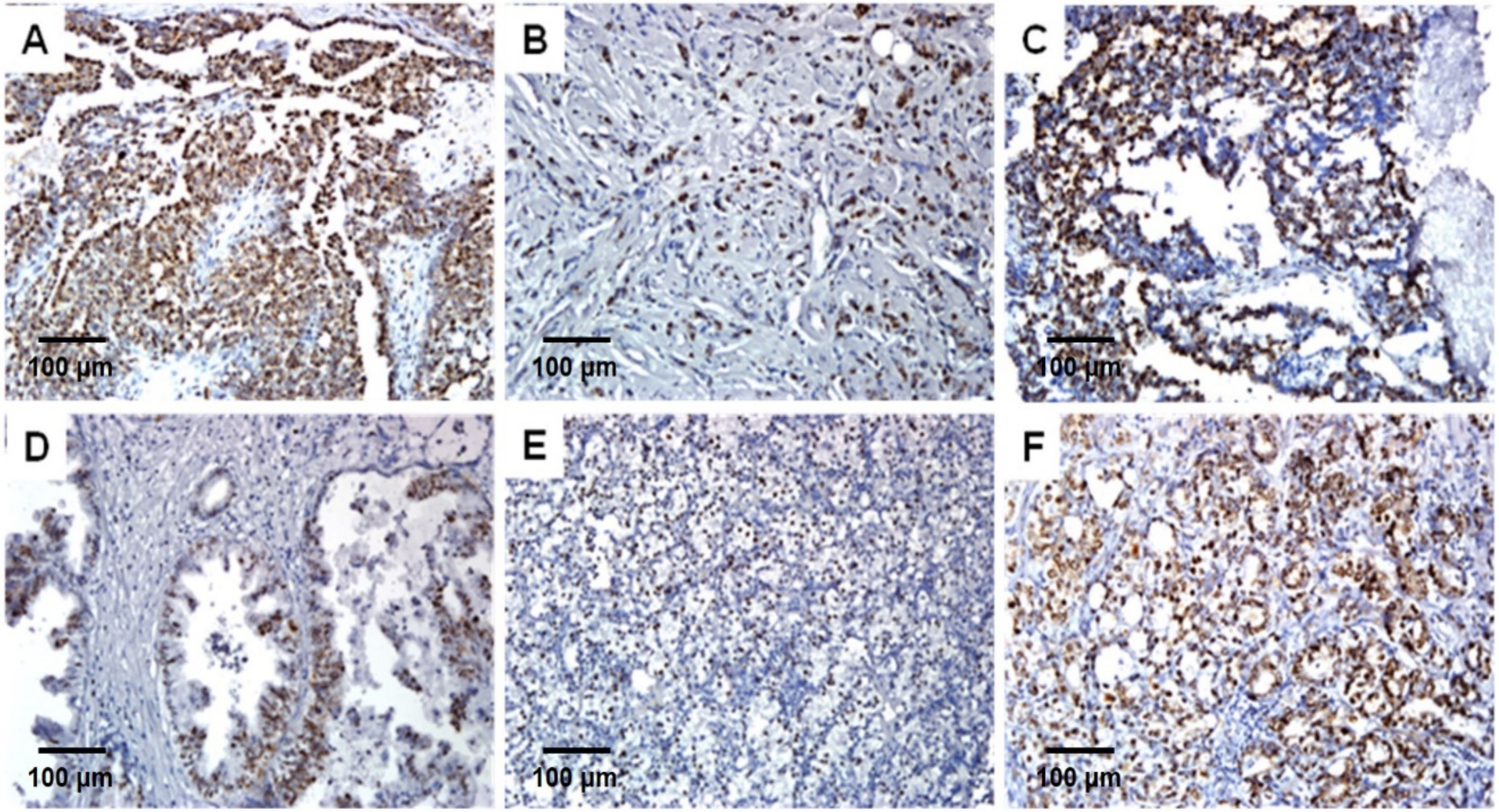

Figure S2. Representative figures of MALAT1 RNA in situ hybridization in A: ovarian high-grade serous carcinoma (HGSC), B: Breast invasive carcinoma of no special type, C: Lung adenocarcinoma, D: Cholangiocarcinoma, E: Renal cell carcinoma, F: Prostate adenocarcinoma**.**

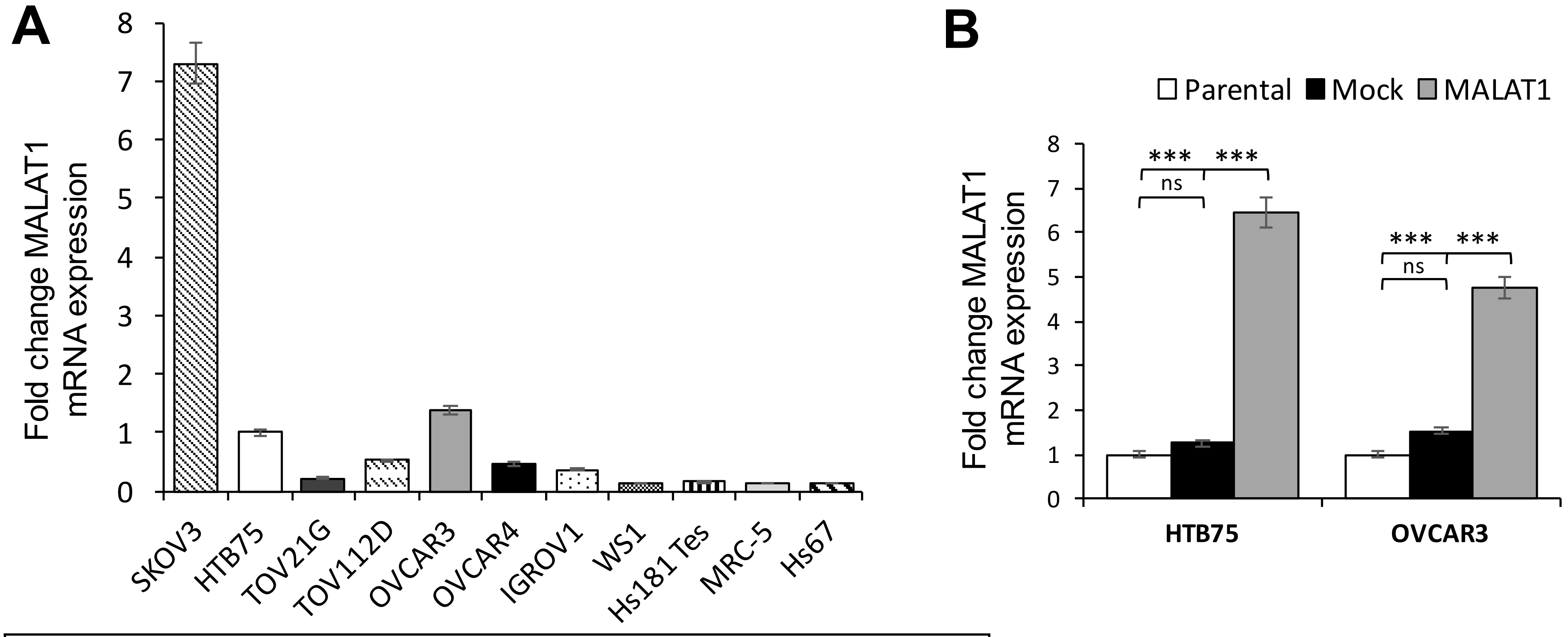

SKOV3	HTB75	TOV21G	TOV112D	OVCAR3	OVCAR4	IGROV1	WS1	Hs181 Tes	MRC-5	Hs67
7.304	1	0.220	0.529	1.393	0.468	0.366	0.137	0.160	0.133	0.145

Figure S3. MALAT1 expression in cell lines. (A) MALAT1 mRNA levels were detected in 7 EOC cell lines and 4 normal human cell lines by RT-qPCR analysis. Relative levels of MALAT1 were normalized by the expression levels in HTB75. **(B)** Effect of MALAT1-transduced overexpression in HTB75 and OVCAR3 cells and mRNA levels were examined by RT-qPCR analysis. Mock-transduced (Mock) and non-transduced (Parental) were used as negative and blank controls, respectively. Representative data are shown from at least 3 independent experiments with the mean ± SD, $***p < 0.001$.

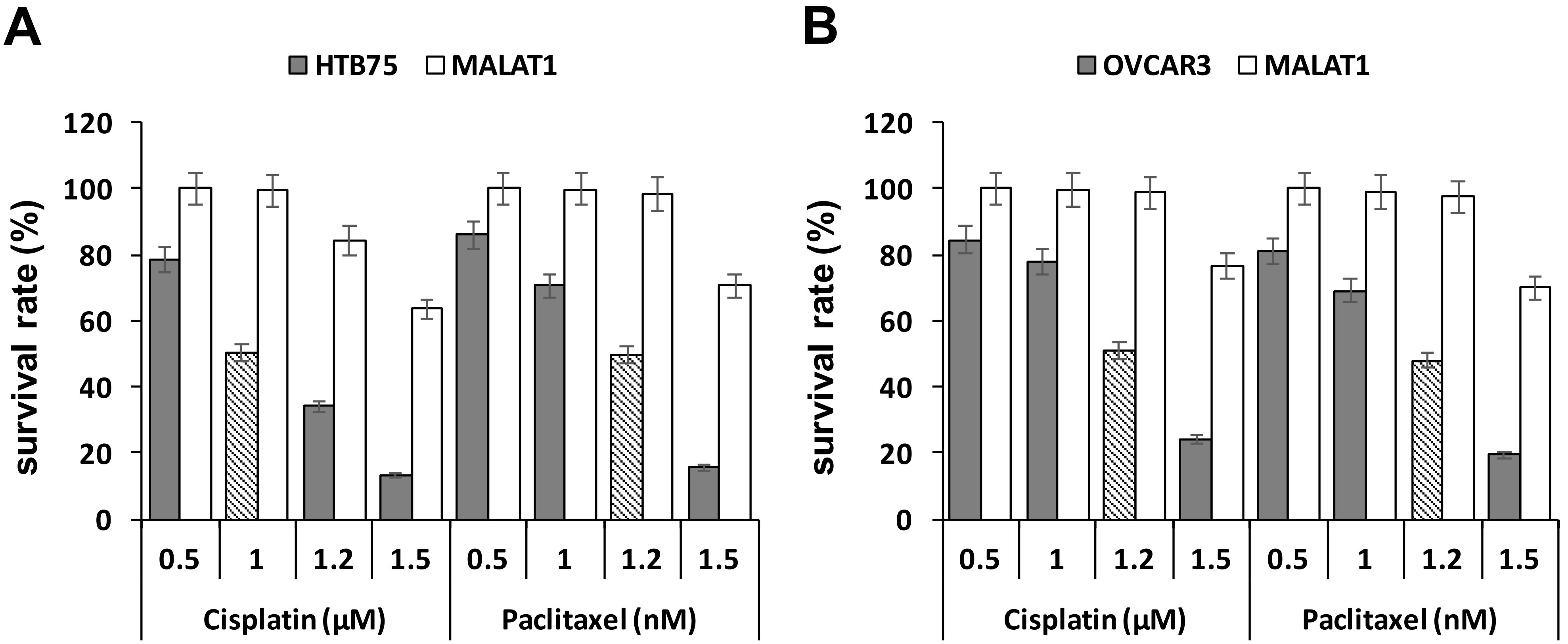

Figure S4. Growth inhibition of cisplatin (CDDP) and paclitaxel (PTX) in HTB75/OVCAR3 parental cells (HTB75/OVCAR3) and HTB75/OVCAR3 cells transduced with MALAT1 (MALAT1). Both CDDP and PTX were tested in the concentration ranges of 0.5-1.5 uM and 0.5-1.5 nM, respectively. The growth inhibition effect was considered significant when the inhibition rate was around 50% (IC50). The IC50 of CDDP and PTX in **(A)** HTB75 and **(B)** OVCAR3, respectively, are shown by slashed bars. Data shown were acquired from three independent experiments.
